# High predation of native sea lamprey during spawning migration

**DOI:** 10.1038/s41598-020-62916-w

**Published:** 2020-04-09

**Authors:** Stéphanie Boulêtreau, Laurent Carry, Elise Meyer, Damien Filloux, Olivier Menchi, Vincent Mataix, Frédéric Santoul

**Affiliations:** 1 EcoLab, Université de Toulouse, CNRS, Toulouse, France; 2 MIGADO, Le Passage, France; 3 EDF, Développement Durable, 4 rue Claude Marie Perroud, 31096 Toulouse, France

**Keywords:** Conservation biology, Freshwater ecology, Invasive species

## Abstract

Sea lamprey (*Petromyzon marinus*) is a unique jawless vertebrate among the most primitive of all living vertebrates. This migratory fish is endangered in much of its native area due to dams, overfishing, pollution, and habitat loss. An introduced predator, the European catfish (*Silurus glanis*), is now widespread in Western and Southern European freshwaters, adding a new threat for sea lamprey migrating into freshwater to spawn. Here, we use a new prototype predation tag coupled with RFID telemetry on 49 individuals from one of the largest sea lamprey European populations (Southwestern France) to quantify the risk of predation for adult sea lampreys during its spawning migration in rivers with large populations of European catfish. We found that at least 80% of tagged sea lampreys (39 among 49) were preyed upon within one month, and that 50% of the released lampreys were rapidly consumed on average 8 days after tagging. This very high predation rate suggests that the European catfish represents a supplementary serious threat of extirpation for the native sea lamprey population we studied. This threat is likely to happen throughout most of the native lamprey distribution area, as the European catfish is becoming established almost everywhere the sea lamprey is.

## Introduction

Freshwater ecosystems are among the most threatened ecosystems worldwide, and the species inhabiting them are declining more rapidly compared to those living in terrestrial and marine biomes^1,2^. The widespread introduction of non-native fishes in freshwaters is a significant threat for native species that may entail complete extirpation of native populations and/or species^3^. The European catfish (*Silurus glanis*), for instance, is now well established in Western and Southern European freshwaters^4^. This species is native from Eastern Europe and has been introduced in Southwestern Europe during the 19^th^ century for sport fishing and aquaculture^5^. It can measure up to 2.7 m in length and has a documented maximum weight of 130 kg, making it, by far, the largest freshwater species by length and mass in its introduced range^6^. Since catfish establishment, large native fishes including anadromous species have no longer benefited from the size-refuge that protected them against native predators (e.g., pike^7^). The catfish is an opportunistic predator that may feed on a wide range of prey^5,8^ and is able to adapt to new prey sources^9^ and to display trophic specialization through foraging on terrestrial birds by intentional beaching^10^ or on the Atlantic salmon in fishways^11^.

**Figure 1:**
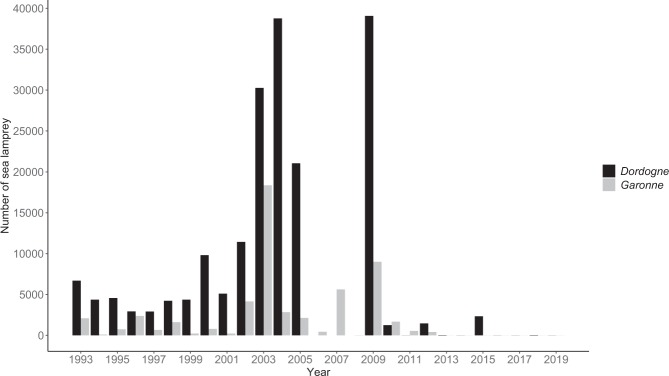
Annual numbers of adult sea lamprey detected by the video fish-counting stations situated in the Dordogne River (black) and the Garonne River (grey) from 1993 to 2019. The Tuillière fishway on the Dordogne River (at 220 km from the confluence between Dordogne and Garonne) and the Golfech fishway on the Garonne River (at around 270 km from the Garonne River mouth) (see Fig. 6) are equipped with permanent video fish-counting stations to monitor fish upstream movements. Numbers in the Dordogne River were not recorded from 2006 to 2008 due to technical problems on the video station. On the contrary, the absence of some bars from 2013 to 2019 on the barplot is not due to technical problems and indicates no effective lamprey passages. A global same temporal pattern was observed in both rivers, with three time periods. From 1993 to 2000, the number of lampreys was quite low, averaging 4,995 and 1,076 individuals per year in the Dordogne and the Garonne, respectively. This number globally increased between 2000 and 2010, reaching records of 18,344 individuals in 2003 in the Garonne and 39,069 in 2009 in the Dordogne. Finally, the annual number experienced a severe drop since 2009: no lamprey has been counted in the Garonne River since 2013 and only 11, 4, 34 and 0 individuals have been counted during the last four years in the Dordogne River.

Lampreys belong to a primitive vertebrate group that diverged approximately 500 Mya^12^. The sea lamprey (*Petromyzon marinus*) is an anadromous species that migrates into rivers to reach the spawning areas. Sea lamprey individuals exhibit no natal philopatry^13^ but regionalization^14,15^, and reproductive adults track pheromones emitted by their stream-dwelling larvae to migrate into rivers^16^. The young larvae burrow in fine sediments, the metamorphosis takes place after several years of larval development, and young adults migrate downstream to the Ocean, where they become parasitic of many marine fish species. The native geographic range of the sea lamprey extends across both sides of the North Atlantic Ocean: along the coast from Labrador to Florida to the West, and from Norway to the Adriatic Sea to the East. The largest native populations are notably observed in the estuaries and rivers of Western Europe, especially in the United Kingdom^17,18^, the Iberian Peninsula^19^, and in France^20^. In these European rivers, lamprey populations have declined over the last 25 years^21^ mainly due to dams, overfishing, pollution, spawning grounds deterioration and changes in water temperature and water quantity associated with climate change^18^. In the two main French sea lamprey populations (i.e. in the Garonne and Dordogne Rivers), the long-term patterns of annual lamprey migrations to spawning areas are concerning, with numbers of returning adult lampreys collapsing since the last decade (Fig. 1).

**Figure 2:**
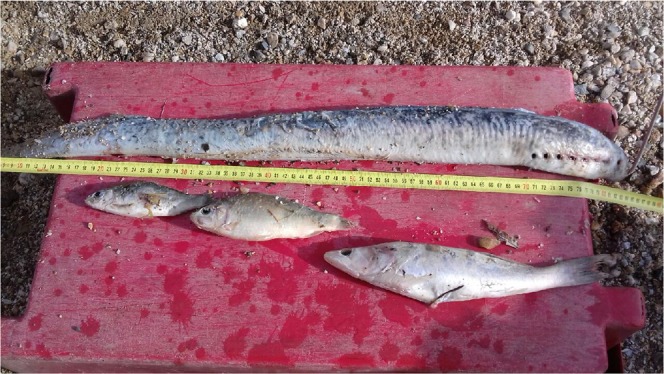
Stomach content of one European catfish individual caught in the Garonne River in April 2019 composed of two Cyprinids, one pike-perch *Sander lucioperca* and one sea lamprey *Petromyzon marinus* (photo credit: Philippe Gauthier, professional fishermen from AAPPED33). We thank Philippe Gauthier for allowing us to use his photograph.

Stable isotope analyses have revealed that the diet of some specialized European catfish individuals could reach more than 50% of marine prey in the Garonne River in Southwestern France^7^. In this river, sea lampreys have also been found in catfish stomach contents (e.g.^22^), and professional fishermen often report the occurrence of sea lamprey individuals in catfish stomach contents (Fig. 2). Despite such evidence, predation on migrating sea lampreys has never been quantified. Direct quantification of catfish predation on migrating sea lampreys using stomach content analyses is tricky, given (i) the high number of sampled catfish required to draw robust conclusions and (ii) the low catchability of large European catfish individuals in large rivers with moderate-to-high water depths such as the Garonne River. Here we quantify the risk of predation to migrating adult sea lampreys during their migration period in one of the largest European sea lamprey populations by combining radio-telemetry and novel acoustic predation tags implanted on adult sea lampreys to track upstream migration and to estimate predation rate.

## Results

Three tagging and tracking experiments (25–50 days in duration) were performed: two in the Dordogne River (named hereafter *Dordogne 1* and *Dordogne 2*) and one in the Garonne River (named hereafter *Garonne*). At the end of the tracking session, 39 lampreys among 49 (80%) were consumed, 2 remained alive, 1 was lost (as never detected) and 7 have unknown status. The proportion of lampreys with unknown status was slightly higher in *Dordogne 2*. However, the proportions of lampreys consumed were similar between experiments *(P* = *0.5581, Fisher’s exact test)*, totalling 21/25 (84%) in *Dordogne 1*, 10/14 (71.5%) in *Dordogne 2* and 8/10 (80%) in *Garonne* after 31 days.

**Figure 3:**
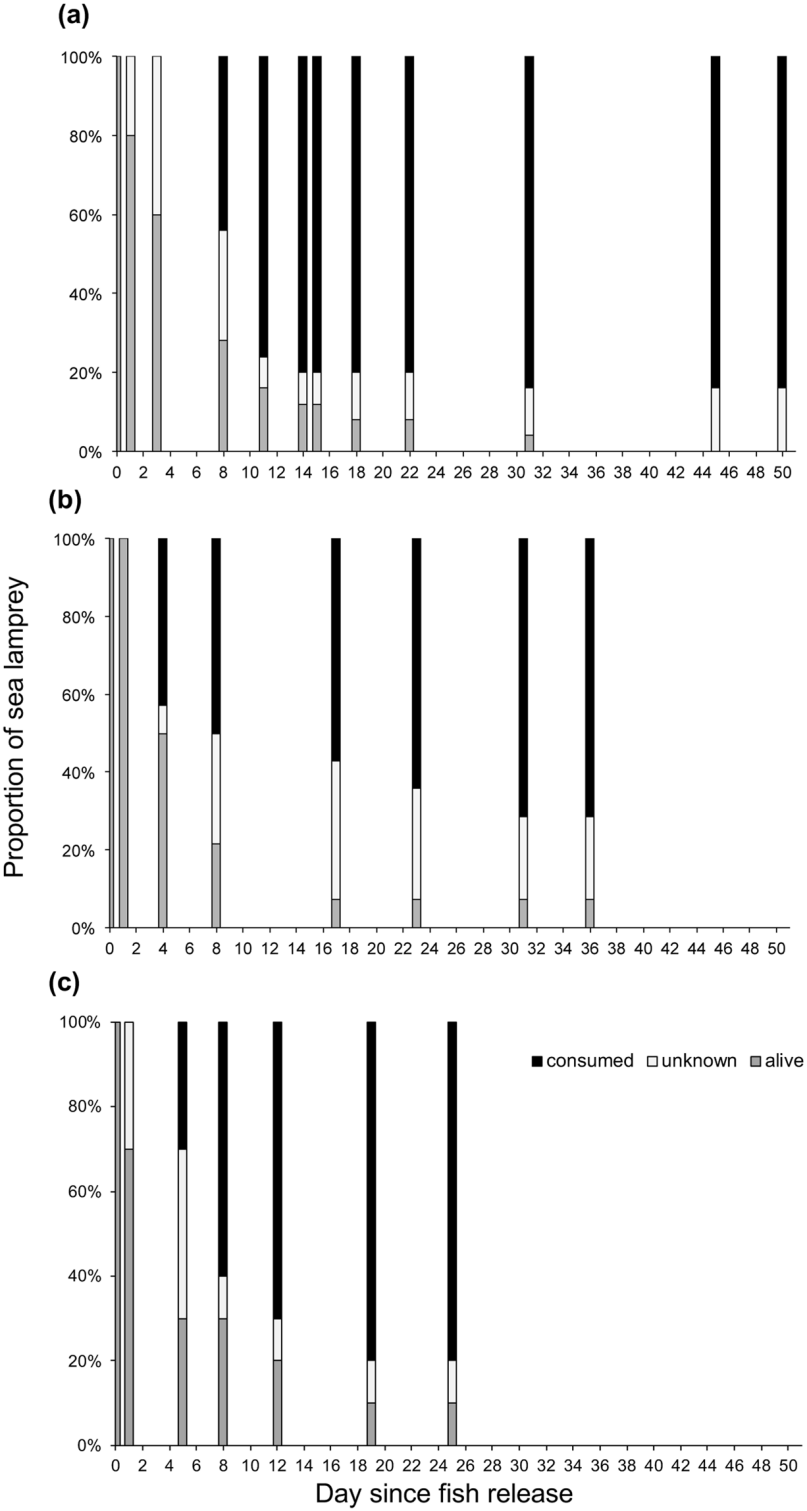
Temporal evolution of the proportion (%) of alive (grey), consumed (black) and unknown-status (white) sea lampreys after their tag and release (day 0): **(a)** in the Dordogne River (experiment *Dordogne 1*, n = 25), **(b)** in the Dordogne River (experiment *Dordogne 2*, n = 14), and **(c)** in the Garonne River (experiment *Garonne*, n = 10).

Each tracking experiment exhibited a similar temporal pattern of predation rate (Fig. 3). First predation events occurred within 2 to 7 days following lamprey release. Nearly 50% of the tagged lampreys (24/49) and 65% of the lampreys with identified status (24/37) were consumed after 8 days. More than 70% of the total lampreys were consumed after 18 days of tracking. Most of the lampreys consumed were detected upstream from the release point; only 2 lampreys were detected downstream from the release point, at 3 and 8 km in *Dordogne 1* and *Dordogne 2* respectively. All lamprey predation events were detected within a stretch of 20-km length, at a mean (±SD) distance of 5.5 (±4.5) km from the release point. Only 4 lampreys among 39 were detected as consumed at more than 10 km from the release point.

## Discussion

**Figure 4:**
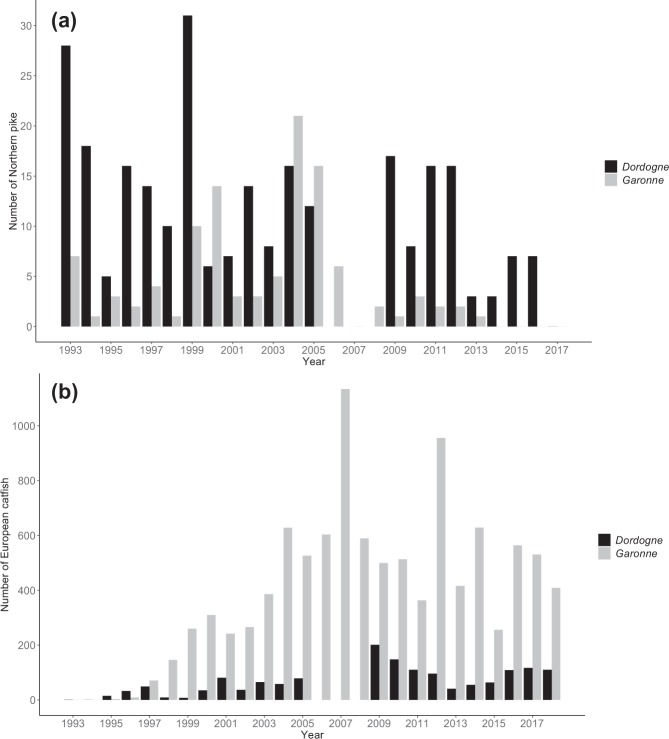
Annual numbers of Northern pike **(a)** and European catfish **(b)** coming back in front of the video fish-counting stations in the Dordogne River (black) and the Garonne River (grey) from 1993 to 2017. The Tuillière fishway on the Dordogne River (at 220 km from the confluence between Dordogne and Garonne) and the Golfech fishway on the Garonne River (at around 270 km from the Garonne River mouth) (see Fig. 6) are equipped with permanent video fish-counting stations to monitor fish upstream movements. Numbers in the Dordogne River were not recorded from 2006 to 2008 due to technical problems on the video station. No other bars on the barplot indicate no passage. (**a**) Annual numbers of pike passages average 4 individuals (±5 SD) and 12 (±8 SD) in Garonne and Dordogne, respectively with maxima of 21 individuals in 2004 in Garonne and 31 individuals in 1999 in Dordogne. (**b**) Catfish passages at the video fish-counting stations occurred in 1995 with 3 and 15 individuals in Garonne and Dordogne, respectively. Those annual numbers progressively increased in both rivers to reach records of 1 134 individuals in 2007 in Garonne and 201 individuals in 2009 in Dordogne. Since those peaks, catfish numbers averaged 520 (±181 SD) individuals in Garonne and 94 (±34 SD) in Dordogne. Such temporal data showed that pike density is far less as catfish density.

The high predation rate we report here (i.e. 80% of the tagged lampreys in both studied rivers) illustrates the high risk of mortality due to predation of adult sea lampreys in Southwestern France. Indeed, thirty-nine among the 49 migrating sea lampreys we tagged were consumed in one month, and this consumption occurred very quickly after lamprey release, with 50% of released lampreys having been consumed on average 8 days after tagging. While we failed in confirming the exact identity of predator, we assumed this predation is mostly due to European catfish. Apart from catfish, the only another predator whose largest specimens may able to consume sea lamprey is Northern pike but the species is very rare in studied rivers (Fig. 4a). Such high predation-related mortality of sea lampreys prior to spawning has been poorly documented in the literature^21^. Mark/recapture and radio-telemetry experiments conducted in two tributaries of the Lake Ontario showed that mortality from predation only explained between 1% and 11% of sea lamprey population reduction, while natural mortality of adult sea lamprey ranged from 6 to 30% during spawning^21^. Another tagging experiment conducted in the River Vouga (Portugal) revealed that adult sea lamprey predation (probably by otters) was largely lower (8% of 25 tagged lampreys) than sea lamprey mortality due to poaching, which reached up to 76%^15^. Our tracking study revealed unprecedented mortality rate estimates for adult sea lampreys due to predation, compared to all previous studies.

Many features could explain why the sea lamprey is heavily consumed by the European catfish during spawning migration. Migrating adult sea lampreys represent a large prey (body length > 85 cm) - compared to other generally smaller fish - that may satisfy the energetic requirements of large European catfish individuals after a low trophic activity period^23^. In Europe, upstream lamprey migration is stimulated by daily water temperature increases at the beginning of spring, also corresponding to catfish activity resumption following winter. The main timings of diel cycle activity are also similar for both species. Adult lampreys undertake nocturnal migrations, moving upstream in freshwaters primarily during dusk and darkness and seeking refuge before dawn. Although some catfish individuals can synchronize their feeding period otherwise^11^, European catfish usually shows feeding activity peaks at night^24^. In addition, lampreys are poor swimmers with weak propulsion capacities, and are known for their ‘burst-and-glide’ intermittent swimming^25^ (contrary to other anadromous species such as Atlantic salmon *Salmo salar*). Atlantic salmon individuals have also been shown to be preyed upon by the European catfish inside a fishway situated in the Garonne River, where the passage of fish is artificially narrow and the probability of predator-prey encounter is high^11^. Nevertheless, the predation rate of 35% (14 salmon on 39) was considerably lower than the predation rate we observed on lamprey, partly due to the higher-speed swimming performances of the salmon compared to sea lampreys, which should facilitate escaping from predators.

Hydrological conditions could also explain why predation-related lamprey mortality was so high and rapid. Indeed, the observed total distance moved before predation varied from 0 to 20 km but was globally low averaging 5.5 (±4.5) km. Migration distance is highly river-flow-dependent. The river flows in Dordogne and Garonne were substantially lower during the tracking experiment compared to average flows measured during the same period in both rivers. Elevated water flow allows sea lamprey to overcome difficult passage stretches^21^ and increases lamprey migratory activity^26^. On the opposite, low flows could have increased their predation success. Indeed, catfish movements have been shown to be inversely related to flow rates^24^. Therefore, observed low flow conditions might have increased the risk for lampreys to be consumed, by both diminishing and increasing the motor activities of the prey (lampreys) and of the predator (European catfish) respectively. It is noteworthy that such water levels have been common in the last decade, and that they are likely to continue.

**Figure 5:**
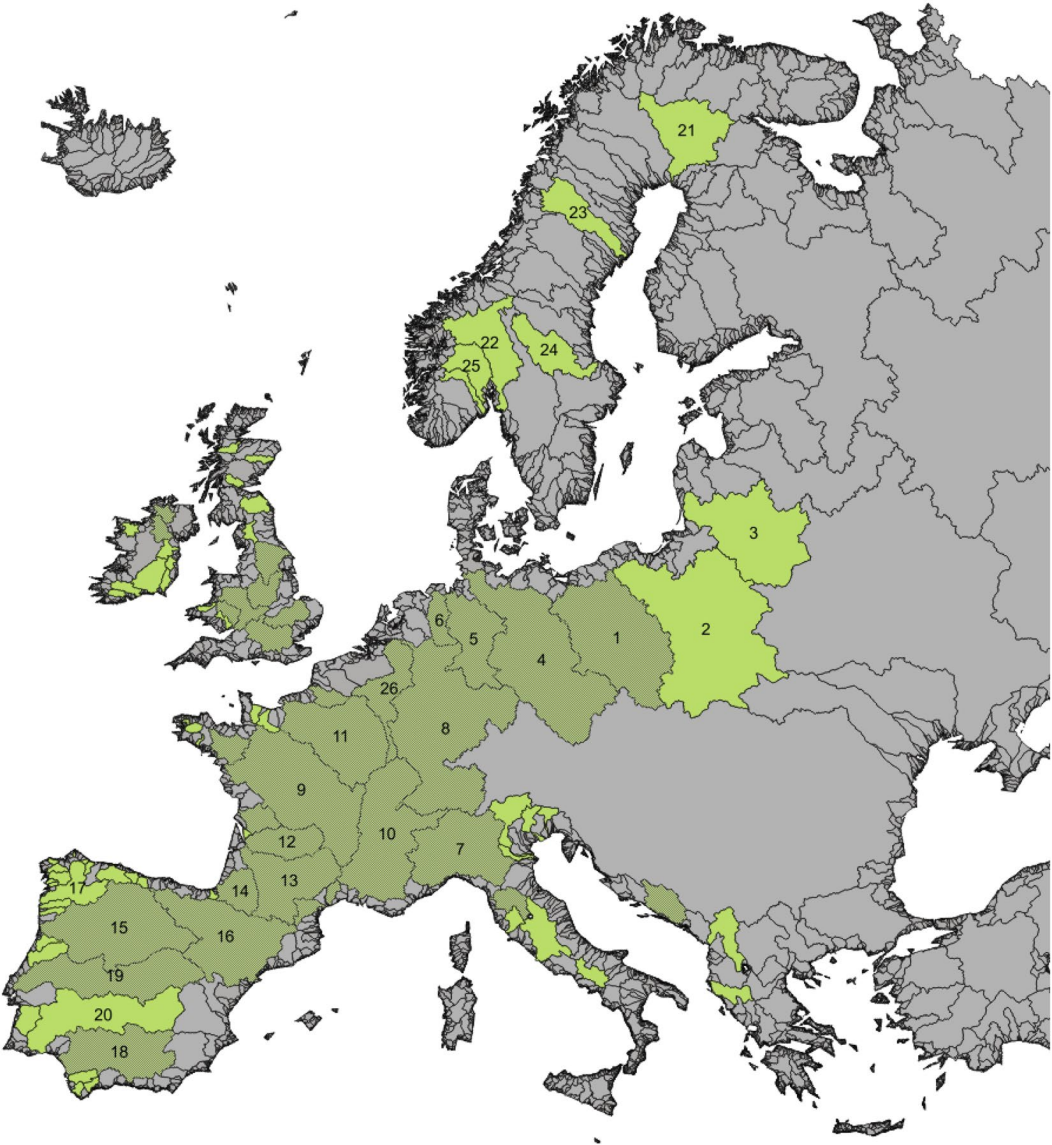
Current distributional range of the sea lamprey in European watersheds. Watersheds where the migrating sea lamprey is reported are displayed in green and watersheds (with an area > 1000 km^2^) where the lamprey co-occurs with the European catfish are displayed in dark green^4^. Sea lamprey distribution map in European watersheds was established from one compilation of the available literature (including scientific reports, books, online data and grey literature). More recent publications are detailed hereafter by watershed: (1), Oder^32^; (2), Wisla^32^; (3), Nemunas^32^; (4), Elbe^33^; (5), Weser^34^; (6), Ems^34^; (7), Pô^35^; (8), Rhine^33^; (9), Loire (http://www.logrami.fr/actions/stations-comptage/); (10), Rhône^36^; (11), Seine^32^; (12), Dordogne (http://www.migado.fr/category/publications/); (13), Garonne (http://www.migado.fr/category/publications/); (14), Adour^33^; (15), Douro^19^; (16), Ebre^19^; (17), Minho^19^; (18), Guadalquivir^19^; (19), Tajo^19^; (20), Guadiana^19^; (21), Kemijoki^36^; (22), Glomma^36^; (23), Umeälven^36^; (24), Dalälven^32^; (25), Skien^32^; (26), Meuse^36^. This distribution was updated for other watersheds with information from^17,37,38^, and the following web sites: http://jncc.defra.gov.uk/ProtectedSites/SACselection/species.asp?FeatureIntCode=S1095; http://www.observatoire-poissons-migrateurs-bretagne.fr/lamproies; http://normandiegrandsmigrateurs.fr/les-poissons-migrateurs-de-normandie/lamproies/comptage-geniteurs-de-lamproies/. The map was generated using QGIS 2.14.0-Essen (https://www.qgis.org/en/site/).

The present experiment demonstrates that predation by the European catfish is a substantial factor of sea lamprey reproductive adult mortality in the Garonne-Dordogne system. This cause of mortality adds to mortality by fishing, as the Garonne-Dordogne system hosts the largest commercial sea lamprey fishery in Europe^27^. Indeed, between c. 50,000 and 90,000 sea lampreys are declared as caught by fishermen upstream from the studied area yearly. Lampreys that successfully escape from fishing have thus to face a massive predation risk to join the spawning area in the Garonne-Dordogne system. The large overlap between sea lamprey and European catfish distribution areas suggest that this cause of adult sea lamprey mortality could also exist in almost all the European sea lamprey distribution area (Fig. 5). Indeed, most of the large watersheds hosting sea lampreys also host the European catfish, except some watersheds situated in Portugal, in Scandinavia, in the North of the United Kingdom, in Poland and in Lithuania.

To conclude, predation by the European catfish has to be added to the long list of factors that seriously threatens native sea lamprey populations (habitat loss and fragmentation, pollution, commercial exploitation, climate change, and water availability^19^). This experiment suggests that the continuous decline and possible extirpation of the largest native population of sea lamprey may occur unless management actions to preserve lampreys and to regulate European catfish are taken.

## Methods

### Study area

The study was conducted upstream of the tidal area of the Gironde estuary (45°02′45.80″ N, 0°36′41.56″ W), where the Dordogne and Garonne rivers discharge (Southwestern France, Fig. 4). The Garonne-Dordogne hydrographic network hosts one of the largest populations of sea lamprey in Europe^20,28^. The Dordogne River runs over 475 km (mean flow = 216 m^3^/s) from its source in the Massif Central to its confluence with the Garonne River. The Garonne River is the largest river of Southwestern France and runs over 580 km (mean flow = 647 m^3^/s) from its source in the Pyrenees to the Atlantic Ocean. The predatory fish guild in both rivers was composed of one native species (the Northern pike *Esox lucius*), and three non-native species (the pikeperch *Sander lucioperca*, the pike *Perca fluviatilis* and the European catfish).

### Sea lamprey tagging and tracking

**Figure 6:**
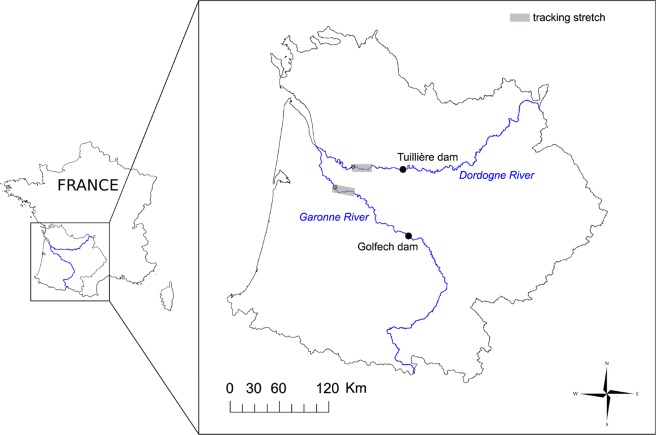
Location of the sea lamprey tracking stretches on Dordogne and Garonne rivers (grey boxes). Black stars indicate lamprey release points. Black circles indicate the localisation of the dams where the permanent video fish-counting stations are installed. The map was generated using QGIS 2.14.0-Essen (https://www.qgis.org/en/site/) and INKSCAPE 0.92.4 (https://inkscape.org).

Sea lamprey tagging and tracking was conducted in two stretches of the Dordogne River (*Dordogne*) and the Garonne River (*Garonne*) to study lamprey survival during the migration period (from March to April 2019) (Fig. 6).

Forty-nine sea lampreys were captured, tagged and released to the river: 39 individuals (mean ± SD total body length = 88 ± 4 cm) in *Dordogne* and 10 individuals (mean ± SD total body length = 82 ± 5 cm) in *Garonne*. We choose to test more individuals on *Dordogne* than on *Garonne* as the population is greatest in the former river. Lamprey capture, tagging and tracking was performed twice in *Dordogne* to mitigate the risk and potential consequences of failure: 25 tagged lampreys were released on March 11^th^ (*Dordogne 1*) and 14 tagged lampreys were released on March 25^th^ (*Dordogne 2*). The manipulation was performed once in the Garonne River on March 21^th^ (*Garonne*).

Lampreys were captured by one professional fisherman using sea lamprey pots at Lamothe-Montravel (44°51′05.09″N, 0°01′36.50″E) and Barsac (44°36′30.98″N, 0°19′00.96″W) for *Dordogne* and *Garonne*, respectively (Fig. 6). Lampreys were then equipped with a 36-mm radio transmitter 1815C (Advanced Telemetry System Inc., NW Isanti, MN, USA), weighing 8 g in air, to follow lamprey movements (e.g. as in^29,15^). Each lamprey was also tagged with an acoustic Vemco tag model V5 (Vemco Ltd., Amirix Systems Inc., Bedford, Nova Scotia, Canada) operating at 180-kHz and with a 143 dB acoustic power output. This ‘predation tag’ is small (4.3 × 5.6 × 12.7 mm) and light (0.65 g in air). It is also equipped with a biopolymer that is digested when conditions are acidic, as it is the case inside predators’ gastrointestinal tracts. Biopolymer digestion leads to a change in the transmitter’s identification number from a pre-predation ID to a post-predation ID (see^30^ for more details).

For each tracking experiment, lampreys were anaesthetized using a benzocaine solution at 10% (0.7 ml/l), measured and tagged. Surgery consisted of a 2–3 cm incision to insert the tags. The incision was closed with 4–5 synthetic absorbable sutures (Vicryl, Ethicon). The procedure took less than 5 minutes. Lampreys were then transferred to a tank filled with river water for at least 15 minutes to recover from anesthesia. Then, they were released in the river inside a cage. The cage was equipped with a small aperture allowing lampreys to find the exit. All individuals rapidly found the exit (i.e. in less than 5 minutes) confirming their good condition and swimming performance.

Tagged lampreys were regularly monitored with receivers embarked on a boat during 25 to 50 days after release, depending on the tracking experiment. Radio-telemetric and acoustic monitoring consisted in traveling along both studied stretches to detect tagged sea lampreys. The *Dordogne* stretch was 32-km long and located between Castillon-la-Bataille (44°51′07.50″N, 0°02′34.80″W; 8 km downstream from the release place) and Le Fleix (44 °C52′32.12″N, 0°14′40.56″E). The *Garonne* stretch was 30-km long and located between Cadillac (44°38′20.91″N, 0°19′19.09″W; 3 km downstream from the lamprey release place) and La Réole (44 °C34′52.49″N, 0°02′28.15″W). Each time one lamprey was radio-tracked with the embarked radio receiver R4500C (Advanced Telemetry Systems Inc., NW Isanti, MN, USA), GPS coordinates were captured and acoustic signals were recorded with an embarked Vemco VR100 acoustic receiver (Vemco Ltd., Amirix Systems Inc., Bedford, Nova Scotia, Canada). Lamprey status was interpreted as alive or consumed depending on the transmitted identification ID. In some cases where a lamprey was not detected with radiotracking or only detected with radiotracking without identifying the acoustic signal, the lamprey status was reported as unknown. As radiotracking was not continuously performed, and because of the lag time due to polymer digestion^30^, the method was not able to precisely inform about the exact location of the predation event. Further, due to the time gap existing between the occurrence of a predation event and its detection, the estimated times of the predation events we present here are likely overestimated, and the distances travelled by lampreys need to be considered with caution.

Each time a lamprey was interpreted as consumed (i.e. reception of a post-predation ID), we tried to capture the predator by angling without success. In some cases, we managed, however, to detect a catfish shape on the echo-sounder screen in the area of the predation signal. While the exact identity of predators was undetermined, predation is likely due to European catfish. Large individuals of another predatory fish inhabiting these rivers may consume adult sea lampreys (i.e. the Northern pike, whose maximal body size can reach 130 cm^31^). Nevertheless, predation by large pikes is probably exceptional, as the abundance of this species is very restricted in both rivers (Fig. 4a) compared to the abundance of European catfish (Fig. 4b). The other reported predator of sea lamprey in freshwaters, the Eurasian otter (*Lutra lutra*)^18^, is absent from the study area.

Observed field conditions in temperature (low temperatures, ranging between 9 to 14 °C in *Dordogne* and between 11 to 14 °C in *Garonne*) and tracking time (7 weeks in *Dordogne* and 3 weeks in *Garonne*) were compatible with conditions required to correctly identify predation events (i.e. short-term deployments and low water temperatures^30^). Concerning water levels, they were low compared to general mean hydrological conditions in March and April in both rivers. In *Dordogne*, mean daily discharges were 238 and 145 m^3^/s in March and April 2019, respectively (http://www.hydro.eaufrance.fr; station Lamonzie-Saint-Martin) whereas mean long-term daily discharges in this station averaged 376 and 330 m^3^/s in March and April (based on 60 years of measures). In *Garonne*, mean daily discharges were 351 and 396 m^3^/s in March and April 2019, respectively (station Tonneins) against averages of 876 and 844 m^3^/s in March and April in this station.

Lamprey tagging was conducted by Migado, an association in charge of monitoring and managing migratory fish species like the sea lamprey. The tagging procedure was in strict accordance with the National Guidelines for Animal Care of the French Ministry of Agriculture (decree n°2013–118) and the EU regulations concerning the protection of animals used for scientic research (Directive 2010/63/EU). The tagging procedure was approved by the ethical committee of the French region “Nouvelle Aquitaine” for fish and birds (C2EA73; authorization #2019022009545607). Previous field tests carried out in 2017 and 2018 using similar radio-transmitters on 116 lamprey individuals confirmed the low impact of the tagging procedure on lampreys.
